# Influence of schooling and age on cognitive performance in healthy older adults

**DOI:** 10.1590/1414-431X20165892

**Published:** 2017-03-23

**Authors:** N.V.O. Bento-Torres, J. Bento-Torres, A.M. Tomás, V.O. Costa, P.G.R. Corrêa, C.N.M. Costa, N.Y.V. Jardim, C.W. Picanço-Diniz

**Affiliations:** 1 Faculdade de Fisioterapia e Terapia Ocupacional, Instituto de Ciências da Saúde, Universidade Federal do Pará, Belém, PA, Brasil; 2 Laboratório de Investigações em Neurodegeneração e Infecção, Instituto de Ciências Biológicas, Universidade Federal do Pará, Belém, PA, Brasil

**Keywords:** Age-related cognitive decline, Primary prevention, Education, Neuropsychological tests, Memory, Neuroscience, CANTAB

## Abstract

Few studies have examined the influence of a low level of schooling on age-related cognitive decline in countries with wide social and economic inequalities by using the Cambridge Automated Neuropsychological Test Battery (CANTAB). The aim of the present study was to assess the influence of schooling on age-related cognitive decline using unbiased cognitive tests. CANTAB allows cognitive assessment across cultures and education levels with reduced interference of the examiner during data acquisition. Using two-way ANOVA, we assessed the influences of age and education on test scores of old adults (61–84 years of age). CANTAB tests included: Visual Sustained Attention, Reaction Time, Spatial Working Memory, Learning and Episodic Memory. All subjects had a minimum visual acuity of 20/30 (Snellen Test), no previous or current history of traumatic brain/head trauma, stroke, language impairment, chronic alcoholism, neurological diseases, memory problems or depressive symptoms, and normal scores on the Mini Mental State Examination (MMSE). Subjects were grouped according to education level (1 to 7 and ≥8 years of schooling) and age (60–69 and ≥70 years). Low schooling level was associated with significantly lower performance on visual sustained attention, learning and episodic memory, reaction time, and spatial working memory. Although reaction time was influenced by age, no significant results on *post hoc* analysis were detected. Our findings showed a significantly worse cognitive performance in volunteers with lower levels of schooling and suggested that formal education in early life must be included in the preventive public health agenda. In addition, we suggest that CANTAB may be useful to detect subtle cognitive changes in healthy aging.

## Introduction

Few studies have examined the influence of low schooling levels on age-related cognitive decline in countries with wide social and economic inequalities. A large proportion of the Brazilian population above 60 years of age have low education levels, with 24.36% being illiterate and 34.85% have no more than 7 years of schooling. Previous studies demonstrate that education can influence performance on cognitive tests, and lower education levels are associated with faster cognitive decline with aging (1,2). Moreover, education may help to decrease normal age-related cognitive decline and neuroprotection may be associated with cognitive reserve (3). These findings highlight the importance of investigating the influences of low schooling levels on cognitive status within the Brazilian population.

Many psychometric procedures are strongly dependent on socio-cultural and educational background (4), and the application of such tests in less educated individuals may result in their misclassification as cognitively impaired (5). Thus, in the present study, we applied an unbiased automated neuropsychological assessment to minimize the possible influence of the experimenter that is associated with traditional pencil-and-paper tests (6). The Cambridge Automated Neuropsychological Tests Battery (CANTAB) is a visuospatial stimulus battery that employs touchscreen technology to obtain non-verbal responses from participants. By using visually attractive stimuli, the CANTAB allows increasing and decreasing the difficulty of a given task, adapting the test to a wide variety of cognitive performances, and maintaining the user's interest during the tests (7).

The CANTAB is considered reliable for assessing type and degree of functional loss and the specificity of aging-associated changes in the temporal and prefrontal lobes (8). It is adequate for cognitive assessment across cultures and education levels, in both longitudinal and cross-sectional studies, with minimal interference of the examiner during data acquisition (9–13). Because CANTAB is reportedly reliable, valid, and specific (14), it was used in our previous exploratory comparative investigation to assess performances of Brazilian healthy young (20-40 years old) and older adults (≥65 years old) on selected CANTAB and language tests to identify possible clusters of cognitive performances. Our prior results showed that age significantly influenced CANTAB and language tests’ scores, and that CANTAB was more sensitive than language tests for detecting subtle cognitive differences between young and aged Brazilian populations (15).

In Brazil, few studies have been done using CANTAB. Previous findings were limited to data from multiple sclerosis patients, Duchenne muscle dystrophy patients and normal children (16,17). To the best of our knowledge, there is no neuropsychological study using CANTAB to assess influence of schooling on age-related cognitive decline in the Brazilian population.

Here, we investigated to what extent age and education influence processing speed, visual episodic memory and working memory in a Brazilian older adult population using unbiased cognitive tests from CANTAB and tested the hypothesis that the effects of age and schooling would interact and aggravate age-related cognitive decline.

## Material and Methods

### Participants

For the present cross-sectional cognitive assessment, 167 old adult volunteers (60**-**84 years of age) were evaluated. Volunteers were of both genders, living independently in the community and fulfilled the inclusion criteria. Each participant completed an extensive assessment of medical history, health, and cognition. Inclusion criteria included minimum visual acuity of 20/30 (Snellen test); no previous or current history of traumatic brain/head trauma, stroke, language disease, chronic alcoholism, neurological diseases, or memory problems; no active infection detected by hematological analysis and clinical signals and no psychiatric illness, including depressive symptoms, screened by the Geriatric Depression Scale and Diagnostic and Statistical Manual for Mental Disorders - Fifth Edition.

All participants showed normal scores on the Mini Mental State Examination (MMSE), with the necessary adjustments for education level for the Brazilian population. Although it is already known that MMSE does not discriminate mild cognitive impairment, each volunteer met the criteria for normal score on MMSE parameters in accordance with the cut-off point established by Bertolucci et al. (18) (1 to 7 years of schooling, 18 points; ≥8 years of schooling, 26 points) and Brucki et al.(19) (mean**±**SD for each schooling group).

This study was approved by the Research Ethics Committee of Hospital Universitário João de Barros Barreto (protocol No. 3955/09), and observed all ethical recommendations in research involving human subjects.

### Cognitive assessment

Spatial working memory, visual sustained attention, learning and memory, processing and psychomotor speed and accuracy were assessed using selected tasks from the CANTAB, performed in the following sequence: Motor Screening Task (MOT), Spatial Working Memory (SWM), Rapid Visual Information Processing (RVP), Paired Associates Learning (PAL), and Reaction Time (RTI). All tests were administered by trained researchers in a single day, under comfortable temperature and noise conditions. The evaluation room was maintained with background lighting conditions in the mesopic range, and stimuli ranged from 0.161 to 3.211 cd/m^2^. On average, tests were completed within 2 h and a brief break was offered to the volunteers between different tests.

Tests were performed on a touchscreen (Dell Flat Panel Model No. E2014Tt, 49.41 cm diagonal display area, maximum resolution of 1600×900 pixels at 60 Hz), following standard procedures in accordance with the user's manual and as previously described (9,14,15,20). CANTAB tests use non-verbal stimuli for cognitive assessment, and include a practice section that does not count towards the final score. The MOT test evaluates an individual's ability to understand simple commands and to correctly use the touch screen, including required finger position and contact pressure to respond to stimuli. The CANTAB tests progress with increasing complexity that adapts to each individual performance. Table 1 shows a detailed description of each test and their respective outcome measurements.

**Table 1:**
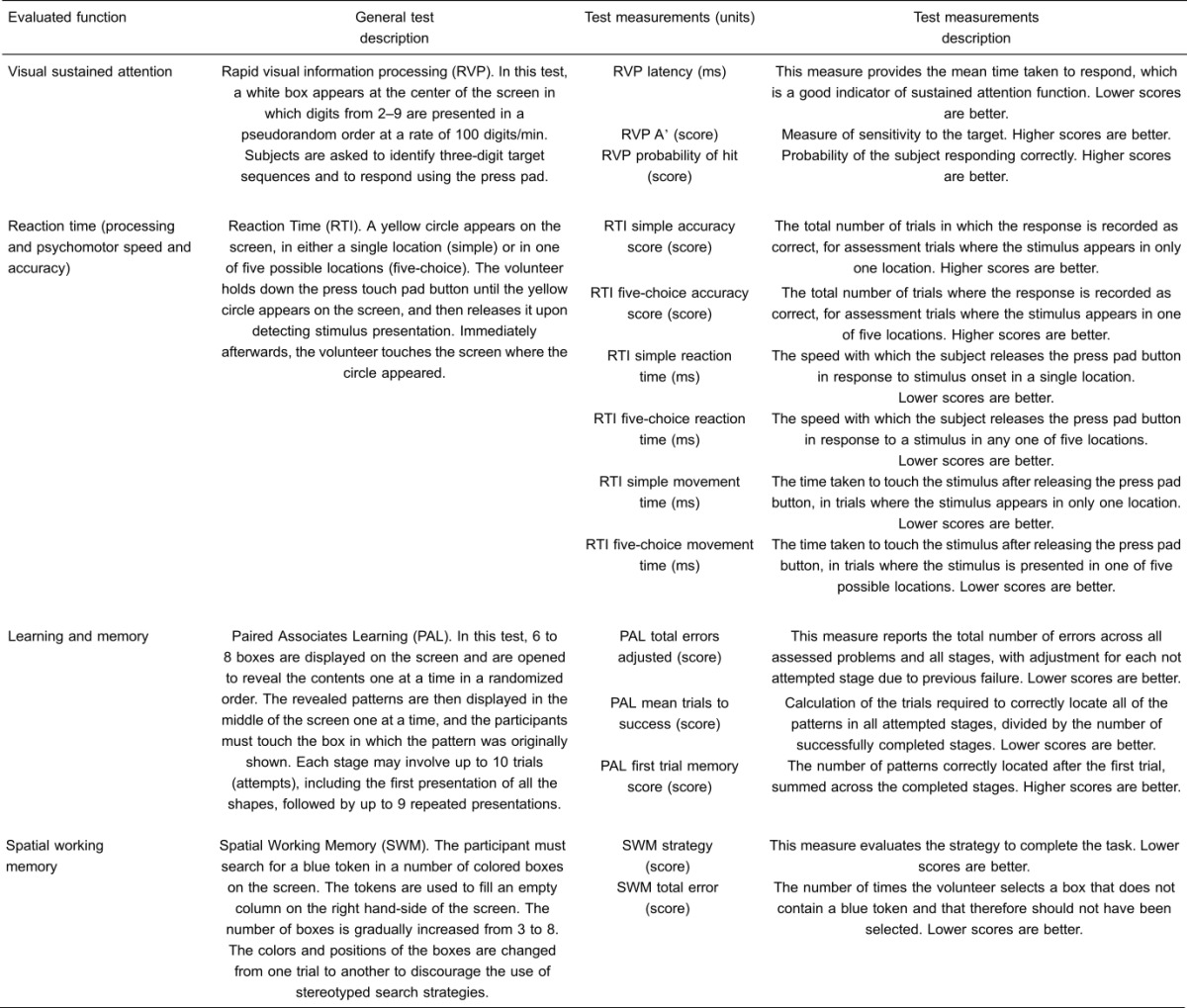
Description of cognitive tests (based on CANTAB user manual).

### Statistical analysis

Prior to statistical analysis, outlier values (based on deviations) were excluded. The statistical significance level was set at P<0.05. Results are reported as means±SE.

To investigate possible interaction between the effects of education and age on cognitive test performance, individuals were grouped by education level (1-7 *vs* >7 years of schooling) and age (60-69 years *vs* 70-84 years), giving a total of four groups: low education <70, low education ≥70, high education <70, and high education ≥70. Two-way analysis of variance and Bonferroni correction *post hoc* tests were used to test the *a priori* hypothesis that years of schooling and aging simultaneously interact and aggravate cognitive decline. Statistical tests were performed using GraphPad Prism¯ (USA).

Pearson analysis and linear regression were used to investigate possible correlations and interdependence between cognitive scores and education level. To that end, we grouped all individuals of different schooling levels and same age group (high+low education level <70 years old or high+low education level ≥70 years old). Similarly, Pearson and linear regression between cognitive scores and age were done by grouping all individuals of different age groups but same schooling level (any age with low education level or any age with high education level).

## Results

The age of the volunteers ranged from 61 to 84 years, with a mean of 71.10±0.39 years. Volunteers had between 1 and 20 years of schooling, with a mean of 8.33±0.33 years. The mean MMSE total score was 27.68±0.14 points. All participants accurately completed the motor screening test, demonstrating adequate sensorimotor ability and comprehension of touchscreen procedures to perform all tests.

### Influence of age and schooling on cognitive assessment

The age and years of schooling of the four groups were: low education <70 group (n=26; 4.15±0.27 years of schooling; 66.92±0.31 years old); low education ≥70 group (n=44; 4.09±0.32 years of schooling; 74.80±0.62 years old); high education <70 group (n=46; 11.34±0.42 years of schooling; 66.20±0.28 years old); and high education ≥70 group (n=51; 11.40±0.42 years of schooling; 74.45±0.49 years old). Table 2 presents the group performances on each cognitive test.

**Table 2:**
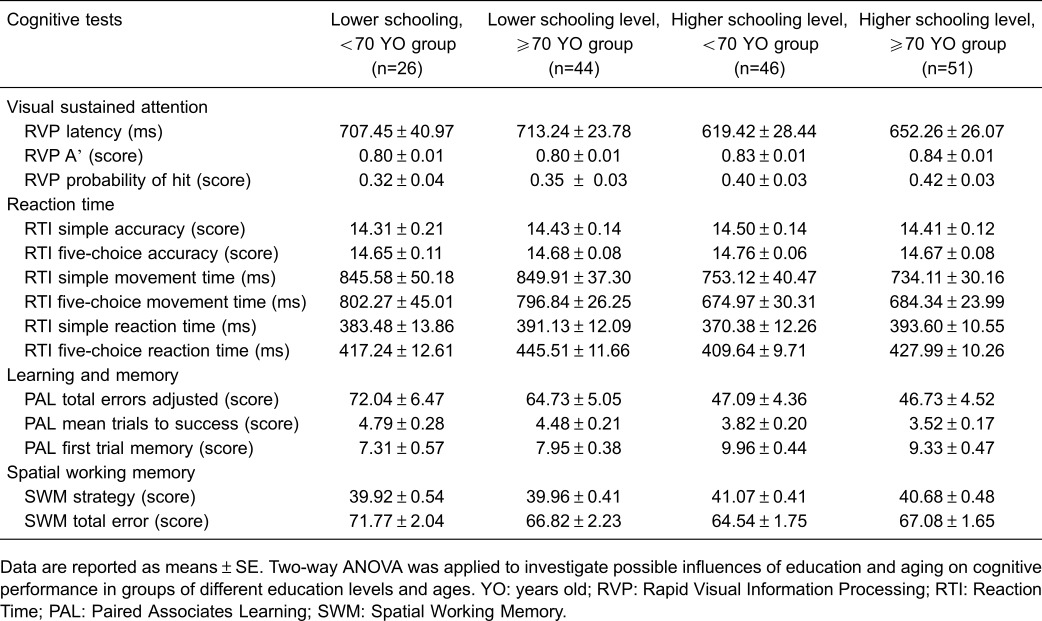
Cognitive performance scores of the studied groups.

**Figure 1:**
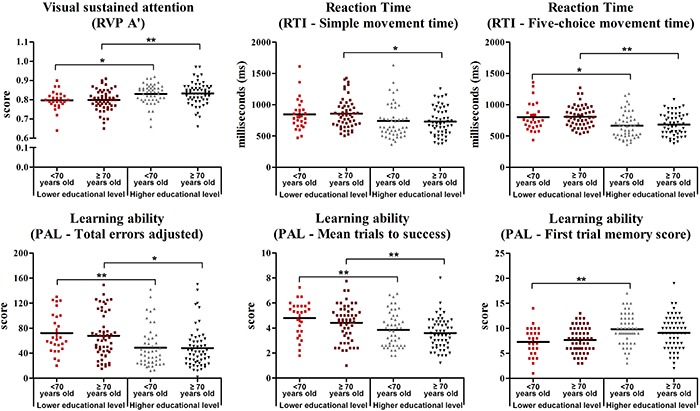
Graphical representations of cognitive performance. Results show the dispersal distribution of cognitive performance test scores in the lower education level <70 years old group (red dots); lower education level ≥70 years old group (dark red dots); higher education level <70 years old group (gray triangles); and higher education level ≥70 years old group (dark gray triangles). Black bars represent the mean score. RVP: Rapid Visual Information Processing; RTI: Reaction Time; PAL: Paired Associates Learning. *P*<*0.05; **P*<*0.01 (two-way ANOVA).

Two-way analysis of variance was applied to each test score. Figure 1 shows individual performance variability on selected cognitive tests and illustrates significant differences in *post hoc* tests after two-way ANOVA. These results revealed a significant influence of education level, but not of age, on all analyzed measurements relating to visual sustained attention (RVP Latency: F=6.34, P=0.013; RVP A′: F=15.19; P=0.0001; RVP PH: F=5.46; P=0.02) and learning and memory functions (PAL TEA: F=17.56, P≤0.0001; PAL MTS: F=20.34, P≤0.0001; PAL FTMS: F=17.68, P≤0.0001). Years of schooling also influenced the Reaction Time scores for the simple and five-choice movement time tests (RTI SMT: F=6.91, P=0.009; RTI 5CMT: F=15.26, P=0.0001) and strategy on the Spatial Working Memory Test (SWM Strategy: F=3.88, P=0.05). No significant interactions were detected between variables.

*Post hoc* analysis indicated that years of schooling influenced cognitive performance on learning and memory measured by PAL FTMS (P≤0.001) of the <70 group but not of the ≥70 group. Schooling also influenced cognitive performances on Reaction Time on five-choice movement latency test for the <70 and ≥70 groups (P≤0.05 and <0.01, respectively), on the PAL total errors adjusted test (P≤0.01 and <0.05, respectively) and PAL mean trials to success test (P≤0.01 and *<*0.01) and on performances on sensitivity to the target on Rapid Visual Information Processing Test (RVP A′: P≤0.05 and <0.01, respectively). In the ≥70 group, education level also affected performances on movement latency on simple paradigm of Reaction Time test (RTI SMT: P≤0.05). RVP latency, RVP PH and SWM Strategy performances were significantly influenced by schooling in the initial analyses, but *post hoc* analysis revealed no significant differences. An isolated influence of age on the RTI five-choice reaction time test (RTI 5CRT: F=4.19; P=0.04) was detected, but no significant result on *post hoc* analysis was found.

**Figure 2:**
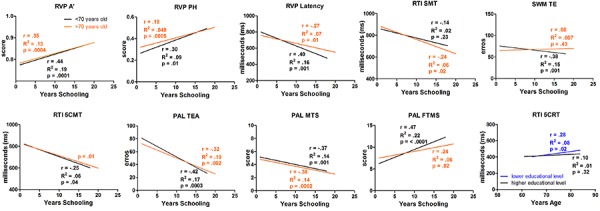
Cognitive performances in different tests as a function of years of schooling. Black lines represent <70 years old subjects and orange lines represent ≥70 years old subjects. Correlation (r) and regression (R^2^) indices and P values are shown for each test and group. RVP: Rapid Visual Information Processing; RVP PH: RVP Probability of Hit; RTI: Reaction Time; RTI SMT: RTI simple movement time; SWM: Spatial Working Memory; SWM TE: SWM total error; RTI 5CMT: RTI five-choice movement time; PAL: Paired Associates Learning; PAL TEA: PAL total errors adjusted; PAL MTS: PAL mean trials to success; PAL FTMS: PAL first trial memory score; RTI 5CRT: RTI five-choice reaction time.

Significant linear correlations and regressions were detected between education level and cognitive performance (Figure 2). Analysis pointed out significant correlations between performance and schooling for the <70 groups on RVP Latency, RVP A′, RVP PH, RTI 5CMT, PAL TEA, PAL MTS, PAL FTMS and SWM TE and for ≥70 groups on RVP Latency, RVP A′, RVP PH, RTI SMT, RTI 5CMT, PAL TEA, PAL MTS and PAL FTMS. Inter-group analysis showed a significant correlation between performance and age for the low education groups on RTI 5CRT.

Linear regression analysis pointed out significant interdependence between education level and performance on cognitive tests for <70 groups on RVP Latency, RVP A′, RVP PH, RTI 5CMT, PAL TEA, PAL MTS, PAL FTMS and SWM TE and for ≥70 groups on RVP Latency, RVP A′, RVP PH, RTI SMT, RTI 5CMT, PAL TEA, PAL MTS, PAL FTMS. Significant regression index was detected between performance and age for the lower education groups on RTI 5CRT. Full details of correlation and regression analysis are shown in Supplementary Tables S1-S4.

## Discussion

The present study aimed to measure the influence of schooling level and age on cognitive performance among Brazilian volunteers by using CANTAB tests. We further used two-way ANOVA on the cognitive test performances to test the hypothesis that the influences of age and schooling would interact and aggravate age-related cognitive decline. Our results showed that schooling and aging did not interact within our sample, but that subjects with lower education level had worst performances on tests of visual sustained attention, reaction time, and learning abilities functions. We also identified an isolated effect of age on five-choice Reaction Time, with the two ≥70 groups showing lower performances than the two <70 groups, but these differences were not significant in *post hoc* analysis. These findings suggest that less education in early life is a risk factor for age-related cognitive decline, with a much stronger influence than age itself. Our results are in agreement with previous findings in aging Brazilian populations using other cognitive tests, such as word memory, verbal fluency, and trail making test B (5). The overall evidence suggests that lower education may contribute to impairment of cognition with aging, supporting the importance of schooling early in life as a long-term preventive strategy to reduce age-related cognitive decline. The association found between low education and weak cognitive performance supports the idea that higher education levels may contribute to an increased cognitive reserve. Moreover, our findings confirmed our previous observations that CANTAB improves the signal-to-noise ratio of cognitive assessment (15), expanding these findings to older adult populations.

To our knowledge, this is the first study in Brazilian elderly adult volunteers to demonstrate the influence of education level on performances in CANTAB selected cognitive tests. Overall, our present data provide evidence that CANTAB is an unbiased cognitive assessment tool for measuring educational influences on cognition. The utilized tests included the SWM, which activates the frontal cortex (21,22) by measures of working memory and strategy based on an individual's ability to retain, manipulate, and remember spatial information according to the context; the RVP test of sustained attention, which activates the frontostriatal circuits (23,24); the RTI test, which evaluates the information processing and psychomotor speed following presentation of a single (simple choice) or multi-position (five choice) visual stimulus, and requires complex chain responses and cognitive processing into frontoparietal functions and subcortical areas that regulate the beginning, planning, and execution of the motor action; and the PAL test, which assesses learning abilities and episodic memory, and relies on the integrity of middle temporal (hippocampal) and frontal functions (25,26). The PAL test effectively distinguishes episodic memory in healthy older adults whereas all other cognitive tests uses multidimensional cognitive processing (26).

Previous limitations related to the use of CANTAB have been pointed out suggesting that higher schooling levels may require better cognitive tests to identify subtle differences between individuals (26). However, in the present report using CANTAB, our analyses demonstrated that lower schooling level is associated with slower information processing speed (RVP and RTI), which has an important impact on cognitive aging and may reflect white matter damage and functional connectivity impairments (27,28).

Previous findings using CANTAB and neuroimages showed that reduced episodic memory is associated with diminished hippocampal volume, with reciprocal influences among neuroanatomical and cognitive variables (29). Episodic memory (PAL) relies on hippocampal formation integrity, and higher hippocampal volume is associated with higher cognitive reserve (27). However, because we did not use neuroimages to correlate neuroanatomical changes with CANTAB results, whether age-related episodic memory decline is explained by hippocampal volume reduction in association with lower schooling remains to be elucidated.

It must also be considered that learning and memory decline among older adults is reduced after exposure to multisensory and cognitive stimulation (enriched environment) (30–33). Specifically, De Oliveira et al. (31 [Bibr B32]) examined stimulation programs applied to older adults living in the impoverished environment of Brazilian long-term care institutions. They reported that persons with lower schooling levels showed significantly improved cognitive test performances after 48 sessions of multisensory and cognitive stimulation program, performed twice a week for 6 months. Thus, besides genetic- and epigenetic-induced brain changes that occur in healthy elderly, we and others (34) suggest that experience-dependent brain plasticity is preserved in non-demented older adults, independent of their education level, encouraging the employment of multisensory and cognitive intervention programs in healthcare policies among aging populations.

Taken together our findings suggest that CANTAB is sensitive for subtle differences on cognitive performance of older adults with low or high education levels, and therefore it is clinically useful for cognitive assessment and early diagnosis of cognitive changes, reducing scale compression, and ceiling and floor effects. Furthermore, early schooling may delay and reduce the age-related cognitive decline.

## Supplementary material

Click here to view [http://bjournal.com.br/supplementary_material/5892.pdf.

## References

[1] Santos NC, Costa PS, Cunha P, Portugal-Nunes C, Amorim L, Cotter J (2014). Clinical, physical and lifestyle variables and relationship with cognition and mood in aging: a cross-sectional analysis of distinct educational groups. Front Aging Neurosci.

[2] Lenehan ME, Summers MJ, Saunders NL, Summers JJ, Vickers JC (2014). Relationship between education and age-related cognitive decline: a review of recent research. Psychogeriatrics.

[3] Jones RN, Manly J, Glymour MM, Rentz DM, Jefferson AL, Stern Y (2011). Conceptual and measurement challenges in research on cognitive reserve. J Int Neuropsychol Soc.

[4] Fitzpatrick AL, Rapp SR, Luchsinger J, Hill-Briggs F, Alonso A, Gottesman R (2015). Sociodemographic Correlates of Cognition in the Multi-Ethnic Study of Atherosclerosis (MESA). Am J Geriatr Psychiatry.

[5] de Azeredo Passos VM, Giatti L, Bensenor I, Tiemeier H, Ikram MA, de Figueiredo RC (2015). Education plays a greater role than age in cognitive test performance among participants of the Brazilian Longitudinal Study of Adult Health (ELSA-Brasil). BMC Neurol.

[6] Smith PJ, Need AC, Cirulli ET, Chiba-Falek O, Attix DK (2013). A comparison of the Cambridge Automated Neuropsychological Test Battery (CANTAB) with "traditional" neuropsychological testing instruments. J Clin Exp Neuropsychol.

[7] Sahakian BJ, Owen AM (1992). Computerized assessment in neuropsychiatry using CANTAB: discussion paper. J R Soc Med.

[8] de Rover M, Pironti VA, McCabe JA, Acosta-Cabronero J, Arana FS, Morein-Zamir S (2011). Hippocampal dysfunction in patients with mild cognitive impairment: a functional neuroimaging study of a visuospatial paired associates learning task. Neuropsychologia.

[9] Robbins TW, James M, Owen AM, Sahakian BJ, McInnes L, Rabbitt P (1994). Cambridge Neuropsychological Test Automated Battery (CANTAB): a factor analytic study of a large sample of normal elderly volunteers. Dementia.

[B10] Robbins TW, James M, Owen AM, Sahakian BJ, Lawrence AD, McInnes L (1998). A study of performance on tests from the CANTAB battery sensitive to frontal lobe dysfunction in a large sample of normal volunteers: implications for theories of executive functioning and cognitive aging. Cambridge Neuropsychological Test Automated Battery. J Int Neuropsychol Soc.

[B11] De Luca CR, Wood SJ, Anderson V, Buchanan JA, Proffitt TM, Mahony K (2003). Normative data from the CANTAB. I: development of executive function over the lifespan. J Clin Exp Neuropsychol.

[B12] Lee A, Archer J, Wong CK, Chen SH, Qiu A (2013). Age-related decline in associative learning in healthy Chinese adults. PLoS One.

[13] Smith PJ, Need AC, Cirulli ET, Chiba-Falek O, Attix DK (2013). A comparison of the Cambridge Automated Neuropsychological Test Battery (CANTAB) with "traditional" neuropsychological testing instruments. J Clin Exp Neuropsychol.

[14] Rabbitt P, Lowe C (2000). Patterns of cognitive ageing. Psychol Res.

[15] Soares FC, de Oliveira TC, de Macedo LD, Tomás AM, Picanço-Diniz DL, Bento-Torres J (2015). CANTAB object recognition and language tests to detect aging cognitive decline: an exploratory comparative study. Clin Interv Aging.

[16] Roque DT, Teixeira RAA, Zachi EC, Ventura DF (2011). The use of the Cambridge Neuropsychological Test Automated Battery (CANTAB) in neuropsychological assessment: application in Brazilian research with control children and adults with neurological disorders. Psychol Neurosci.

[17] Teixeira R, Antunes A, Zachi EC, Roque DT, Taub A, Ventura DF (2011). Memory span measured by the spatial span tests of the Cambridge Neuropsychological Test Automated Battery in a group of Brazilian children and adolescentes. Memory span measured by the spatial span tests of the Cambridge Neuropsychological Test Automated Battery in a group of Brazilian children and adolescentes. Dement Neuropsychol.

[18] Bertolucci PH, Brucki SM, Campacci SR, Juliano Y (1994). [The Mini-Mental State Examination in a general population: impact of educational status]. Arq Neuropsiquiatr.

[19] Brucki SM, Nitrini R, Caramelli P, Bertolucci PH, Okamoto IH (2003). [Suggestions for utilization of the mini-mental state examination in Brazil]. Arq Neuropsiquiatr.

[20] Simpson EE, Maylor EA, Rae G, Meunier N, Andriollo-Sanchez M, Catasta G (2005). Cognitive function in healthy older European adults: the ZENITH study. Eur J Clin Nutr.

[21] Goldman-Rakic PS (1987). Circuitry of the frontal association cortex and its relevance to dementia. Arch Gerontol Geriatr.

[22] Owen AM (1997). The functional organization of working memory processes within human lateral frontal cortex: the contribution of functional neuroimaging. Eur J Neurosci.

[23] Sahakian BJ, Downes JJ, Eagger S, Evenden JL, Levy R, Philpot MP (1990). Sparing of attentional relative to mnemonic function in a subgroup of patients with dementia of the Alzheimer type. Neuropsychologia.

[24] Chari G, Shaw PJ, Sahgal A (1996). Nonverbal visual attention, but not recognition memory of learning, processes are impaired in motor neurone disease. Neuropsychologia.

[25] Persson J, Kalpouzos G, Nilsson LG, Ryberg M, Nyberg L (2011). Preserved hippocampus activation in normal aging as revealed by fMRI. Hippocampus.

[26] Lenehan ME, Summers MJ, Saunders NL, Summers JJ, Vickers JC (2015). Does the Cambridge Automated Neuropsychological Test Battery (CANTAB) Distinguish Between Cognitive Domains in Healthy Older Adults?. Assessment.

[27] Whalley LJ, Staff RT, Fox HC, Murray AD (2016). Cerebral correlates of cognitive reserve. Psychiatry Res.

[28] Madden DJ, Bennett IJ, Song AW (2009). Cerebral white matter integrity and cognitive aging: contributions from diffusion tensor imaging. Neuropsychol Rev.

[29] Persson N, Ghisletta P, Dahle CL, Bender AR, Yang Y, Yuan P (2016). Regional brain shrinkage and change in cognitive performance over two years: The bidirectional influences of the brain and cognitive reserve factors. Neuroimage.

[30] de Macedo LD, De Oliveira TC, Soares FC, Bento-Torres J, Bento-Torres NV, Anthony DC (2015). Beneficial effects of multisensory and cognitive stimulation in institutionalized elderly: 12-months follow-up. Clin Interv Aging.

[31] De Oliveira TC, Soares FC, De Macedo LD, Diniz DL, Bento-Torres NV, Picanço-Diniz CW (2014). Beneficial effects of multisensory and cognitive stimulation on age-related cognitive decline in long-term-care institutions. Clin Interv Aging.

[B32] Lenehan ME, Summers MJ, Saunders NL, Summers JJ, Ward DD, Ritchie K (2015). Sending Your grandparents to university increases cognitive reserve: the Tasmanian Healthy Brain Project. Neuropsychology.

[33] Then FS, Luck T, Luppa M, König HH, Angermeyer MC, Riedel-Heller SG (2015). Differential effects of enriched environment at work on cognitive decline in old age. Neurology.

[34] Volkers KM, Scherder EJ (2011). Impoverished environment, cognition, aging and dementia. Rev Neurosci.

